# Evidence for intermolecular forces involved in ladybird beetle tarsal setae adhesion

**DOI:** 10.1038/s41598-021-87383-9

**Published:** 2021-04-08

**Authors:** Naoe Hosoda, Mari Nakamoto, Tadatomo Suga, Stanislav N. Gorb

**Affiliations:** 1 Surface & Adhesion Science Group, Research Center for Structural Materials, National Institute for Materials Science, Namiki 1-1, Tsukuba, Ibaraki 305-0044 Japan; 2 Department of Precision Engineering, School of Engineering, The University of Tokyo, Hongo 7-3-1, Bunkyo-ku, Tokyo, 113-8656 Japan; 3 Collaborative Research Center, Meisei University, Hodokubo 2-1-1, Hino, Tokyo 191-8506 Japan; 4 Department of Functional Morphology and Biomechanics, Zoological Institute at the University of Kiel, Am Botanischen Garten 1-9, 24098 Kiel, Germany; 5 Present Address: East Japan Railway Company, Tokyo, Japan

**Keywords:** Biophysics, Zoology

## Abstract

Why can beetles such as the ladybird beetle *Coccinella septempunctata* walk vertically or upside-down on a smooth glass plane? Intermolecular and/or capillary forces mediated by a secretion fluid on the hairy footpads have commonly been considered the predominant adhesion mechanism. However, the main contribution of physical phenomena to the resulting overall adhesive force has yet to be experimentally proved, because it is difficult to quantitatively analyse the pad secretion which directly affects the adhesion mechanism. We observed beetle secretion fluid by using inverted optical microscopy and cryo-scanning electron microscopy, which showed the fluid secretion layer and revealed that the contact fluid layer between the footpad and substrate was less than 10–20 nm thick, thus indicating the possibility of contribution of intermolecular forces. If intermolecular force is the main physical phenomenon of adhesion, the force will be proportional to the work of adhesion, which can be described by the sum of the square roots of dispersive and polar parts of surface free energy. We measured adhesion forces of ladybird beetle footpads to flat, smooth substrates with known surface free energies. The adhesive force was proportional to the square-root of the dispersive component of the substrate surface free energy and was not affected by the polar component. Therefore, intermolecular forces are the main adhesive component of the overall adhesion force of the ladybird beetle. The footpads adhere more strongly to surfaces with higher dispersive components, such as wax-covered plant leaves found in the natural habitat of ladybird beetles. Based on the present findings, we assume ladybird beetles have developed this improved performance as an adaptation to the variety of plant species in its habitat.

## Introduction

Many insects are capable of attaching to and walking on flat substrates, where claws cannot contribute to mechanical interlocking^1,2^. For example, ladybird and leaf beetles have adhesive pads on the sole (tarsus), consisting of adhesive tarsal setae, which allow them to attach and walk on flat, smooth substrates. The shape and mechanical properties of adhesive tarsal setae have been previously investigated^3,4^. The adhesive tarsal setae in ladybird beetles have a length of several tens of microns, and the leading edge has a part that contributes to contact formation, adhesion, and friction with a size of approximately several microns to 10 µm, and has a lanceolate-like, a pointed, a spatula-like, or a discoidal shape^4,5^. The Young’s modulus of the setae along their length is not uniform: 6.8 GPa at the bases of the setae and 1.2 MPa at the tip^6^. The two seta-covered tarsomeres (tarsal segments) in the ladybird beetles exhibit different types of setae^5^. Since the soft tarsal terminal is easily deformable, it is adaptable to the adherend surface and plays an important role in establishing/forming the contact (adhesive) area^7^. It is known that the adhesive strength depends on the direction of external forces: When the legs are moved towards the body, the adhesive strength becomes stronger, and when the legs are moved in the opposite direction, the adhesive strength becomes weaker^8^. The adhesion is reversible and quick peeling ensures less energy expenditure during detachment^8^. The setae are covered with fluid secretion^9,10^. Adhesion is greatly affected by the amount of the secretion^8^. It has been reported that a large amount of secretory fluid significantly reduces the adhesive strength^8^. The composition of the secretory fluid varies depending on the insect species. In the case of the ladybird beetle, lipids are the main component^11^. The forces which contribute to adhesion depend on the thickness of the liquid layer^12^. Thus, the knowledge about properties of this secretory fluid and the thickness of the liquid layer in the contact area between the footpad and substrate is essential for understanding the principles of adhesion in such biological systems. In a thicker layer, capillary forces dominate, due to surface tension, whereas in a layer which is sufficiently smaller than the size of the solid surface sandwiching the liquid, the Laplace pressure becomes dominant^12^. When the thickness of the fluid layer is further reduced (less than 10 nm), intermolecular interactions with the substrate surface become possible^13^.

On flat, smooth substrates, the adhesion is stronger when the fluid secretion layer is thinner^12^. Thus, the presence of a thick layer of secretion in some insects was thought to be an adaptation to adhere less strongly to substrates^14^ and/or to fill substrate irregularities to enhance adhesion on rough substrates^15^. The possibility of beetle adhesion being mainly driven by intermolecular interactions was suggested by Stork (1980) for leaf beetles *Chrysolina polita* L.^2^, but little conclusive experimental evidence has been reported due to the difficulty of experimentation with live animals and in situ visualization of fluid layers.

In this study, inverted optical microscopy, scanning electron microscopy and cryo-scanning electron microscopy (cryo-SEM) was used to investigate and determine the thickness of the secretory fluid layer between the substrate surface and ventral surface of the spatulate terminal of the ladybird beetle *Coccinella septempunctata* L. (Coleoptera, Coccinellidae). To evaluate the contribution of intermolecular forces to the beetle tarsal adhesion, traction force measurements of beetles on flat, smooth substrates with different surface free energies were conducted.

In order to evaluate the physical phenomenon (intermolecular force or capillary force) that governs the adhesion mechanism, a theoretical formula was used. The experimental data were plotted versus calculated data to examine whether it was linear relationship. In order to evaluate the effectiveness of this method, an adhesion experiment was conducted with a polymer material. Two types of adhesion experiments were performed: dry contact in which intermolecular force is dominant and wet contact in paraffin oil in which capillary force is dominant. The main component of the secretory fluid of the ladybird beetle *Coccinella septempunctata* L. is 9-methylheptacosane^11^ of a kind of alkane. Liquid paraffin oil (a type of alkane) was used for the wet contact experiment in this study.

## Results and discussion

### Contact area of setal terminals and secreted fluid

**Figure 1:**
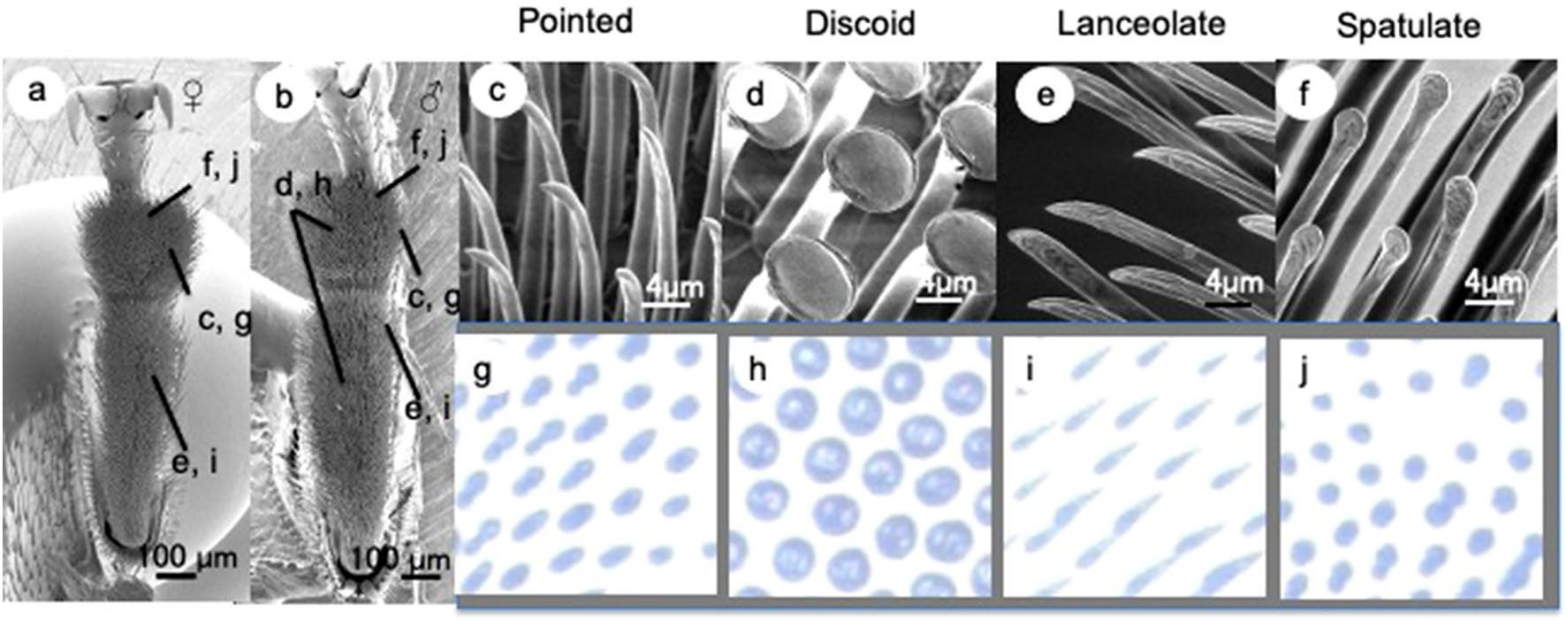
Scanning electron micrographs showing the ventral aspect of female and male tarsi in the seven-spotted ladybird beetle, *Coccinella septempunctata*. Tarsi of (**a**) female and (**b**) male. Four types of tenent setae: (**c**) pointed; (**d**) discoid; (**e**) lanceolate; (**f**) spatula. (**g**–**j**) Optical microscope images of different types of adhesive setae in contact, visualized for a living beetle attached to a smooth transparent polystyrene substrate.

Figure 1a,b show SEM images of the attachment setae on the ventral surface of the adhesive setae in the adult beetle *C. septempunctata*. This beetle has different types of setal terminal shape: spatulate, lanceolate, and pointed in both sexes, and discoid only in males. The corresponding contact areas of different setae observed by optical microscopy are shown in Fig. 1c–j.

**Figure 2:**
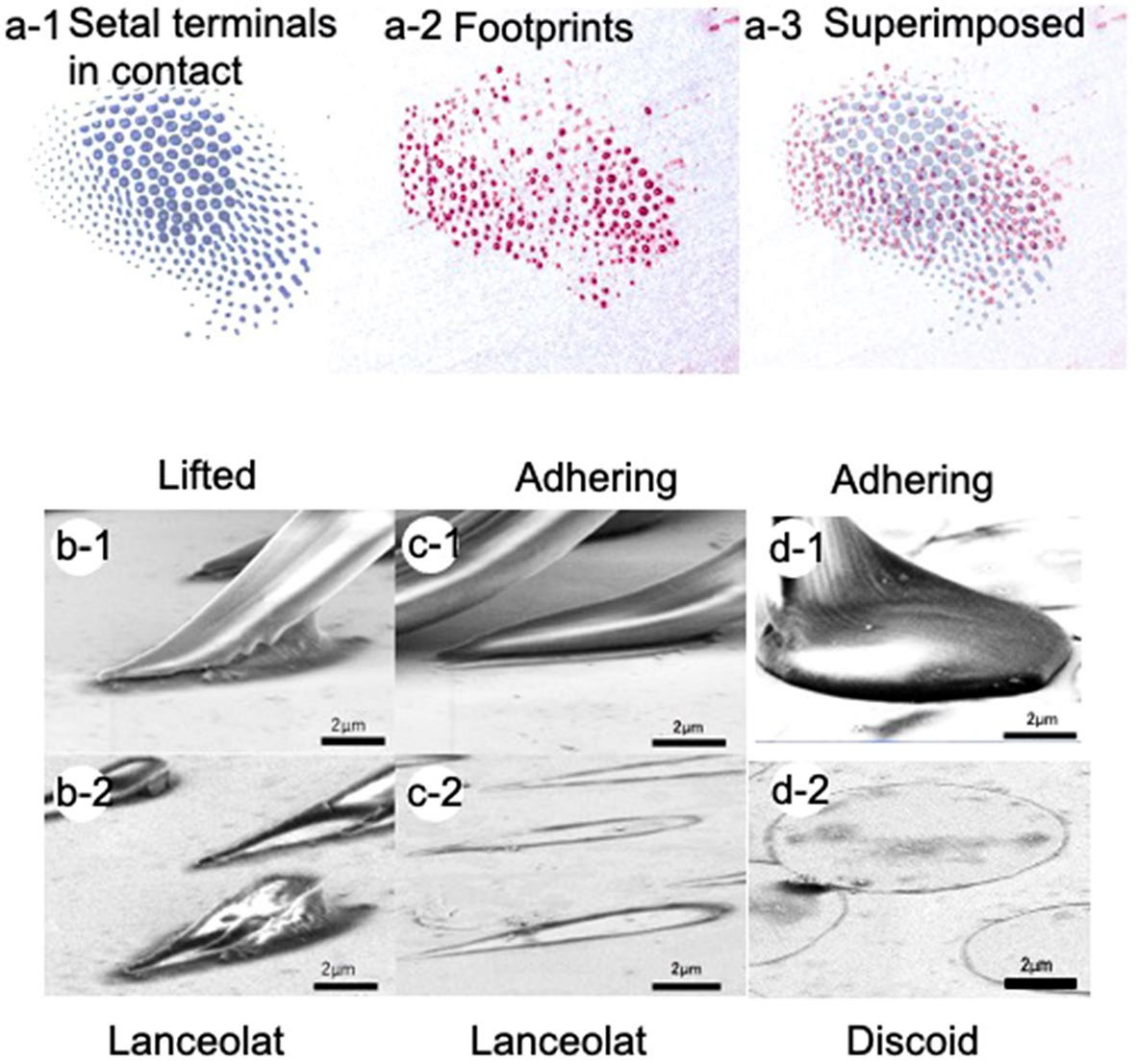
(**a**) Adhesive setae in contact with polystyrene substrate observed by optical microscope and (**b**–**d**) setae and secretory fluid observed by cryo-SEM. (**a-1**) Contact area of tenent setae by optical microscope. (**a-2**) Secretory fluid remaining after beetle foot removal. (**a-3**) Discoid setae left less amount of remaining secretory fluid than other types. (**b**–**d**) Cryo-SEM of setae and secretory fluid of male *C. septempunctata* on AuPd-coated glass. (**b-1**) Lanceolate seta slightly lifted. (**c-1**) Lanceolate and (**d-1**) discoid setae tightly adhering to the substrate. (**b-2**,**c-2**,**d-2**) Frozen fluid remaining after setae removal in (**b-1**,**c-1**,**d-1**), respectively.

**Figure 3:**
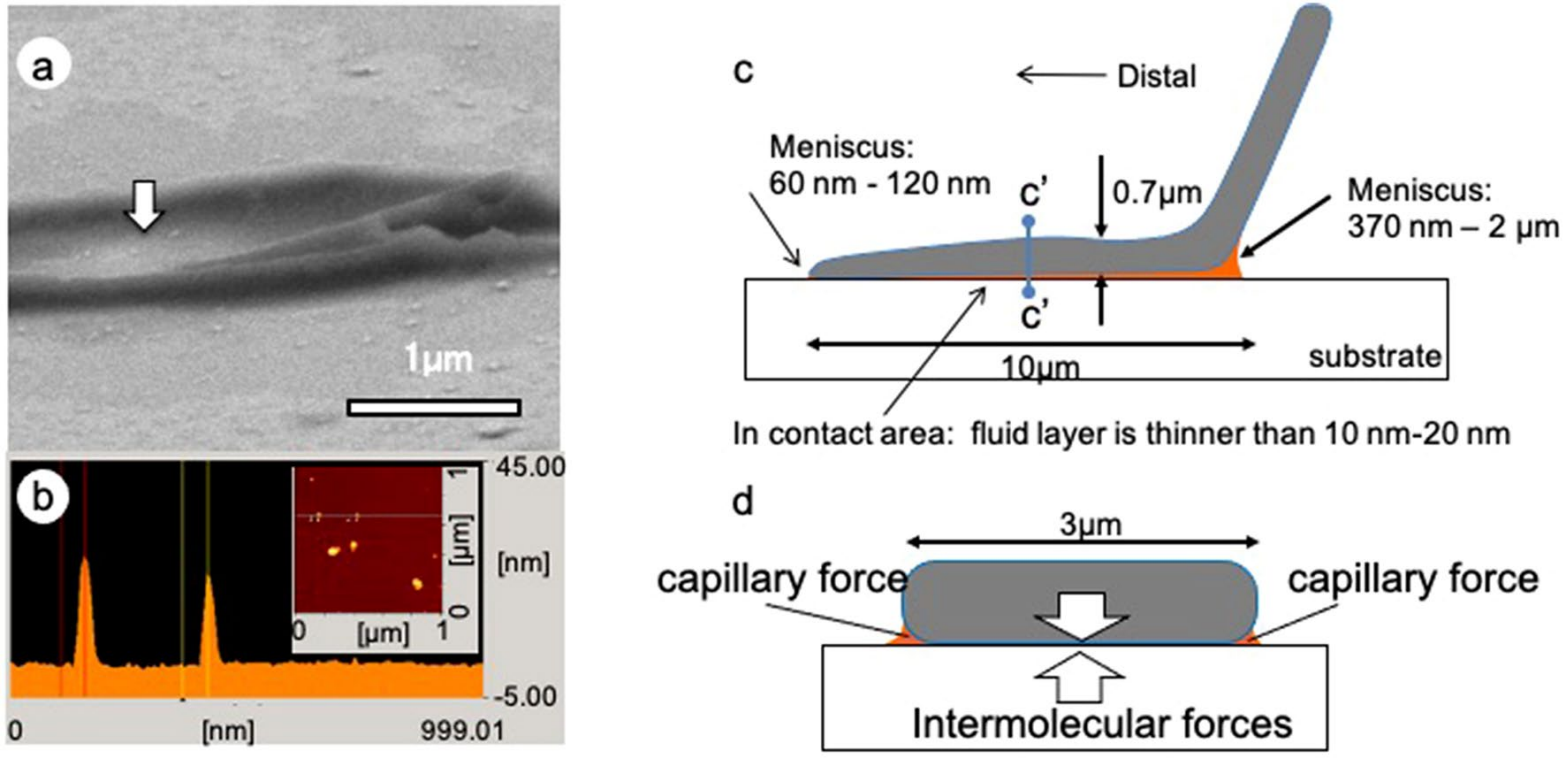
(**a**) Cryo-SEM micrograph of frozen secretory fluid of lanceolate setae on glass substrate with AuPd thin layer. (**b**) AFM image of the glass substrate. The arrow in (**a**) indicates an AuPd particle with a height of 10–20 nm, which indicates that the layer thickness of the secretory fluid is less than 10–20 nm. (**c**) Schematic view of the distribution of secretory fluid between the ladybird beetle setal lanceolate and the substrate. (**d**) Front view of the cross section of the c’–c’ line in (**c**). Arrows indicate the force acting between the setal lanceolate and the substrate. The thin arrow indicates capillary force (Laplace pressure), and the thick arrow indicates intermolecular force.

Footprints of the secretory fluid remain on the substrate surface after the beetle has walked on it. Figure 2a1–a3 shows the contact areas of setal tips while in contact (Fig. 2a-1), the secretory fluid remaining on the substrate after the setae were removed (Fig. 2a-2), and superimposed images (Fig. 2a-3). We coloured these areas blue and red, respectively. The results show that the contact area of discoid setae released less tarsal fluid than the spatulate ones. Thus, the amount of tarsal fluid left as footprints differs depending on the setal type.

### Cryo-SEM of secretory fluid

To illustrate how the secretory fluid is distributed within the contact area between the beetle setae and substrate, the contact region was observed by using cryo-SEM (Fig. 2b-1,c-1,d-1. The cryo-SEM micrographs of male setae in contact with a glass substrate coated with a 3-nm-thick layer of AuPd show that the secretory fluid on the substrate frozen at − 120 °C after removal of the foot remained as a meniscus at the setae-substrate interface (Fig. 2b-2,c-2,d-2). The meniscus was thicker at the proximal (towards the body) edge (370–2000 nm) and thinner at the distal edge (approximately 100 nm). At the bottom of the terminal contact element, the layer of secretory fluid was exceptionally thin. To examine the thickness of the secretory fluid in this region, the glass substrate coated with AuPd was observed by atomic force microscopy (AFM). There were small protrusions of 9.3 nm ± 0.4, mean ± SE, n = 5 and 19.4 nm ± 4.2, mean ± SE, n = 5 in height, which can be seen as small raised dots in Fig. 3a, b. These protrusions were also present in the image at the glass-setal terminal interface in Fig. 3a. The height of the protrusions allowed us to estimate the thickness of the secretory fluid, which was markedly less than the height of the protrusions.

Based on these results, the distribution of the secretory fluid under the lanceolate terminal contact element of the setae is shown in Fig. 3c,d. There was a notable difference in the meniscus between the distal and proximal parts of the lanceolate tip. This difference in meniscus size may generate a difference in negative pressure (Laplace pressure) in the fluid around the meniscus, because Laplace pressure is proportional to the reciprocal of the size of the meniscus^12^. Therefore, the distal part of the setal terminal with a thin layer of fluid of 50 nm will exhibit a 40 times greater force than the proximal part of the setal terminal having a thicker amount of 2 µm. As a result, the proximal part, where contact breakage begins during detachment, can be more easily separated from the substrate than the distal part.

### Ladybird beetle traction forces on various flat, smooth substrates

**Figure 4:**
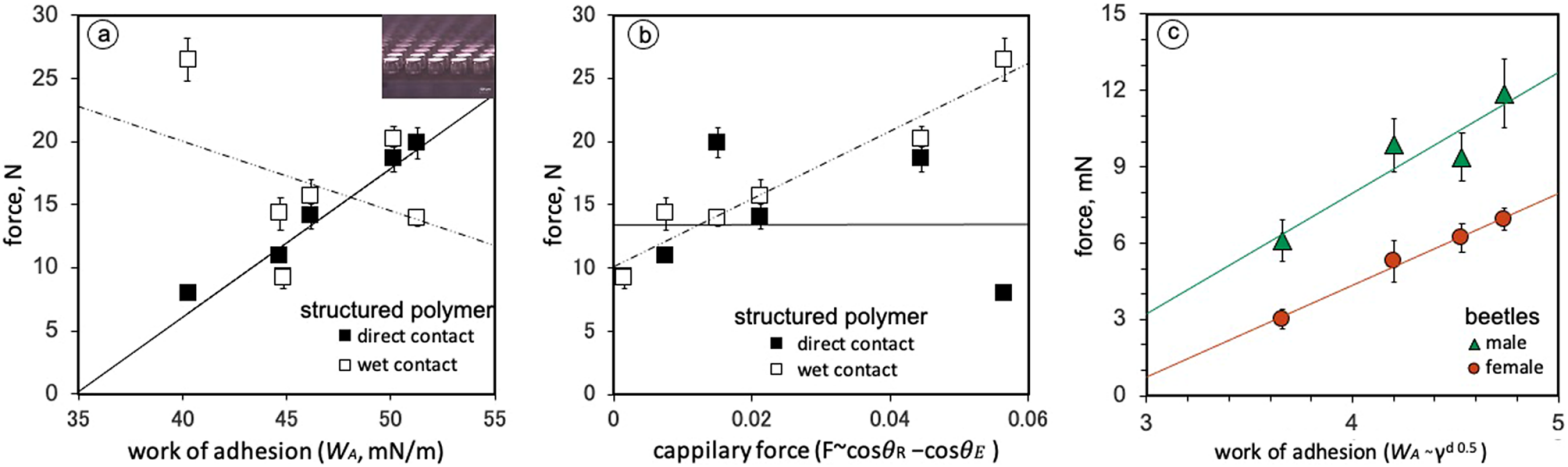
(**a**,**b**) show the results of the pull-off force of structured polymer (PDMS) from the substrates with different surface energies. The black square symbols show the direct contact of the polymer (dry contact) to the substrate. The white square symbols show a polymer coated with liquid paraffin and then contacted to the substrate (wet contact). The pull-off forces were plotted in (**a**) versus work of adhesion *W*_*A*_ which was calculated by Eq. (1) and data in Table 2a. Error bars show the standard error. The inset in (**a**) illustrates the structured polymer sample. In the case of dry contact, the forces increased linearly with the work of adhesion. On the other hand, the wet contact had no linear relationship with the work of adhesion. The same experimental data as (**a**) were plotted in (**b**) versus the capillary force calculated by Eq. (4) and data in Table 3. The forces of the wet contact increased linearly with the capillary force. The dashed line was determined by linear regression y = 266.9x + 10.1 with coefficient of determination (R^2^) of 0.9366. The dry contact polymer samples had no linear relationship with the capillary force. These results indicate that *W*_*A*_ and *F* can be used as indicators to determine the main component of the adhesive force acting during dry and wet contact. The experimental results for the ladybird beetle were plotted versus *W*_*A*_ ~ $\sqrt{{\gamma }^{d}}$, which is a function of adhesive work calculated using data in the Table 2b, in (**c**). The traction force of the ladybird beetle (male: green triangle symbol, female: red circular symbol) increased linearly with the work of adhesion *W*_*A*_. Males have greater adhesive strength than females, but both males and females tended to have the same adhesive properties.

The traction forces of female (*n* = 12) and male (*n* = 12) beetles walking on four different substrates were measured (Fig. 4c). The results showed that the traction forces on substrates with different surface free energies were statistically different (paired t-test, P < 0.05). A detailed data comparison is shown in Table 1. The traction forces of female beetles walking on the substrates were 5.3 ± 0.8 mN (mean ± SE) on glass, 6.2 ± 0.6 mN on Si, 3.0 ± 0.4 mN on resine, and 6.9 ± 0.4 mN on polystyrene EOG. The traction forces of male beetles walking on the substrates were 9.8 ± 1.1 mN (mean ± SE) on glass, 9.4 ± 0.9 mN on Si, 6.1 ± 0.8 mN on resin and 11.9 ± 1.4 mN on polystyrene EOG. Male beetles generated higher traction force than females on any substrate. Both female and male beetles had different traction forces depending on the type of substrate as discussed below.

**Table 1:** Statistical analysis of differences in traction forces of male and female beetles *C. septempunctata* between various substrates compared pair-wise by a paired *t*-test (male *n* = 12, female *n* = 12).

Comparison	*T*	*P*	Statistically significant change	Sex
(a) vs (b)	0.533	0.605		Male
(a) vs (c)	6.667	< 0.001	Yes	Male
(a) vs (d)	− 1.590	0.140		Male
(b) vs (c)	4.277	0.001	Yes	Male
(b) vs (d)	− 2.954	0.013	Yes	Male
(c) vs (d)	− 4.473	< 0.001	Yes	Male
(a) vs (b)	− 1.112	0.290		Female
(a) vs (c)	2.906	0.014	Yes	Female
(a) vs (d)	− 2.798	0.017	Yes	Female
(b) vs (c)	5.963	< 0.001	Yes	Female
(b) vs (d)	− 1.272	0.229		Female
(c) vs (d)	− 6.555	< 0.001	Yes	Female

*t*, *t*-test value; *p*, probability value. (a), Glass; (b), silicon wafer; (c), smooth methacrylate low-viscosity resin; (d), smooth polystyrene treated by EOG.

### Verifying dry and wet adhesion mechanism

When two solids are in direct contact (dry contact), intermolecular forces act between their surfaces. The work of adhesion (*W*_*A*_)^16^ can be described by the following equation:

1 $${W}_{A}=2\left(\sqrt{{\gamma }_{A}^{d}{\gamma }_{B}^{d}}+\sqrt{{\gamma }_{A}^{P}{\gamma }_{B}^{P}}\right)$$

Here, ${\gamma }_{\upbeta }^{d}$ and ${\gamma }_{\upbeta }^{P}$ are respectively the dispersive and polar parts, of the surface free energy for surface β = A or B.

To test the contribution of intermolecular forces to the overall adhesion mechanism, this work employed a polymer (polydimethylsiloxane, PDMS) structure composed of thin plates on top of pillars. This microstructure was tested in contact with six substrates, and pull-off forces were measured by a digital force gauge. The *W*_A_ value was then calculated by using Eq. (1) with the dispersive and polar parts of the surface free energies of the substrates and PDMS. A linear relationship was observed between the pull-off force and *W*_A_ when no fluid was present in the contact area between the PDMS pillars and substrate (Fig. 4a). The linear equation for the dry adhesion was determined by linear regression to be y = 1.1751x − 40.876 with a coefficient of determination (R^2^) of 0.9154. The white square symbols in Fig. 4a indicate the pull-off forces of the structured polymer with a thin layer of paraffin oil. Unlike the case of dry contact, no linear relationship was detected between the pull-off force and *W*_A_, if a thin paraffin oil layer was present.

When two flat solids contact a thin liquid layer (wet contact), the adhesion between the two solids is strongly influenced by the Laplace pressure. When the radius (*R*) of the contact face on the pillar structure with the thin liquid layer is much larger than the distance between the two solid faces (*h*: thickness of the liquid layer), the adhesion can be described by the following equation^12^:

2 $$F\sim \frac{\pi {R}^{2}2\mathrm{\gamma cos}\theta }{h}$$

Here, *γ* is the surface free energy for liquid. *F* is the pull-off force. *θ* is the contact angle of the liquid on the solid substrate. However, this equation describes an equilibrium state. In a dynamic state, a threshold force is required to move the contact line of the three phases with a receding contact angle^17^. In this case, the pull-off force can be described by the following equation:

3 $$F\sim \frac{\pi {R}^{2}2\gamma \left(cos{\theta }_{R}-cos{\theta }_{E}\right)}{h}$$

Here, ${\theta }_{R}$ is the receding contact angle between the substrate and liquid and ${\theta }_{E}$ is the contact angle of the liquid at equilibrium. If the amount of liquid is constant using the same liquid, then $\gamma$ and *h* do not change, so the force is considered to be proportional to $(\mathrm{cos}{\theta }_{R}-\mathrm{cos}{\theta }_{E})$ and can be calculated from the wetting angle and receding angle of paraffin on the substrate, as shown below.

4 $$F\propto (\mathrm{cos}{\theta }_{R}-\mathrm{cos}{\theta }_{E})$$

A linear relationship was observed between the pull-off force and capillary force when fluid was present in the contact area between the PDMS pillars and the substrate (Fig. 4b). In contrast, as indicated by the black square symbols in Fig. 4b, marking the pull-off force of the structured polymer during dry contact, there was no linear relationship between the pull-off force and capillary force *F.*

Using the above results, we can determine whether the mechanism which mainly contributes to insect leg pad adhesion is based on capillary or intermolecular forces (van der Waals force) or both. The traction force exhibited by ladybird beetles was found to be proportional to *W*_*A*_, which is mainly caused by the dispersive component of the substrate surface free energy. This suggests that the ladybird beetle adhesion is caused less by capillary forces from the secretory fluid but rather by intermolecular forces.

With our approach, it is possible to draw a connection between the experimentally measured traction forces experienced by ladybird beetle hairy footpads and *W*_*A*_ from Eq. (1). Figure 4c shows the linear relationship between the traction force and the square root of the dispersive part of the intermolecular force. The green and red lines were determined by linear regression to be y = 4.7381x + 10.988 and y = 3.6044x − 10.077 with coefficients of determination (R^2^) of 0.8645 and 0.9924, respectively. Interestingly, the ladybird beetle tarsal adhesive pads seem to rely mainly on the dispersive component of the surface free energy of the substrate, thus indicating that the tarsal setae and/or the tarsal fluid are composed of a material with a high dispersive component. This result shows that adhesion of the setae of the ladybird beetle relies strongly on intermolecular forces. The liquid did not provide capillary force as the main force for adhesion. Rather, it may contribute to making the setae close adhere to the substrate using meniscus and make it easier to peel off.

In this study, we experimentally proved the main mechanism of ladybird beetle adhesion. From the abovementioned results, we proposed the main mechanism contributing to the adhesion by estimating factors proportional to the measured traction force. In the case of the ladybird beetle, the traction force was proportional to *W*_*A*_. Thus, we conclude that the adhesion of this beetle is caused by intermolecular forces, as previously predicted by Stork (1980)^2^.

Interestingly, the adhesion of ladybird beetle feet was proportional to the square root of the dispersive component of the surface free energy, but the polar component had no effect. The *W*_*A*_ value can be calculated from the intermolecular forces acting at the contacting surfaces and is the sum of the multiplications of the same components of the surface free energy. Therefore, the foot of the ladybird beetle tarsi and/or tarsal fluid should be composed of materials having a low polarity and high dispersive forces. This implies that ladybird beetles are expected to adhere stronger to surfaces with a higher dispersive component of the surface free energy. Such surfaces can be found in ladybird beetle’s natural habitats: e.g., the leaf surfaces of several plant species are covered with amorphous crystalline epicuticular wax layers, which possess a higher dispersive component of the surface free energy^18^. Thus, we assume that the seven-spot ladybird beetles are equipped with tarsal setae and fluid which operate particularly well on various plants in the beetle’s habitat.

## Conclusion

This study experimentally proved that the adhesion mechanism of beetle's footpads, which has been hypothesized for a long time, is based on intermolecular forces.

The attachment forces occurring in ladybird beetle adhesive tarsal setae turned out to be proportional to *W*_*A*_, as predicted by a theoretical model using an adhesion mechanism based on intermolecular forces. This allows us to conclude that the adhesion mechanism of ladybird beetles is based on intermolecular forces. Future investigations on other insect species with this approach would be desirable to prove the generality of this principle.

## Methods

### Beetles

The seven-spotted ladybird beetle, *C. septempunctata*, was used as a model insect species because of its comparatively large size, ease of collection and rearing. Adult ladybird beetles were collected from various plants (*Vicia angustifolia*, *Paeonia suffruticosa,* etc.) in the field in Tsukuba city, Japan (36° 04′ 12″ N, 140°08′ 04″ E). For the observation of the secretory fluid by cryo-SEM, adult seven-spotted ladybird beetles *C. septempunctata* were purchased from the company: Katz Biotech AG, Baruth, Germany. The beetles were kept in small ventilated cages (70 × 70 × 100 mm^3^) at a temperature of 23–25 °C and 38–60% relative humidity, and were fed with 3% sugar solution, which was provided soaked into cotton-wool balls. The photoperiod during the study was 10 h.

### Substrate preparation

Different flat, smooth substrates were prepared for experiments: (a) soda-lime glass (Pre-cleaned micro slide glass, thickness 1.2–1.5 mm, 75 × 52 mm, Matsunami glass Ind., Ltd., Japan), (b) a (100) silicon wafer (P-typ, Rare metallic Co., LTD, Japan), (c) methacrylate low-viscosity resin polymerized at 70 °C for 8 h (Agar Low viscosity resin kit, Agar Scientific Ltd., British), (d) polystyrene EOG (treated by ethylene oxide glycol) (Asnol Sterilization Petri Dish, AS ONE Corporation, Japan), (e) polystyrene, and (f) a sapphire plate. (e) and (f) were used for the pull-off force experiment with the structured polymer. These substrates were chosen because they have different wettability and are readily available. The surface free energies of all substrates were estimated by contact angle measurements using a motion analysis microscope VW-6000 (Keyence, Osaka, Japan) and the Owens, Wendt, Rabel, and Kaelble method^19^ using distilled water, ethylene glycol (99.5%), diiodomethane (97%), glycerol (99%), and n-hexadecane (99%). The laboratory conditions during the contact angle measurements were as follows: a temperature of 21–27 °C and 20–67% relative humidity. Before the force measurements for the pillar structure, the glass substrate and silicon wafer were successively cleaned with acetone, ethanol, and distilled water and dried by using a nitrogen jet. Before the force measurements for the beetles, the glass* substrate was successively cleaned with a mild detergent, tap water and ethanol and dried by using a nitrogen jet. Silicon* wafer was used without cleaning. The surface free energies were summarized in the Table 2(i) and (ii).

**Table 2:** The dispersion part and polar part of the surface free energy on the substrates.

Substrates	Dispersion part, mN/m	Polar part, mN/m
**(i) Substrates for force measurements for the specially-shaped pillar structure**		
Glass	22.6	40.1
Silicon	18.5	42.4
Methacrylate low-viscosity resin	17.7	2.2
Polystyrene EOG	22.5	1.5
Polystyrene	22.7	0.7
Sappire	26.6	14.1
PDMS	21.2	0.3
**(ii) Substrates for force measurements for the beetles**		
Glass*	17.7	17.2
Silicon*	20.5	29.6
Methacrylate low-viscosity Resin	13.4	5.2
Polystyrene EOG	22.5	1.5

Contact angle of the liquid paraffin at equilibrium on the substrates and receding contact angle were measured using a motion analysis microscope. The data were summarized in the Table 3.

**Table 3:** Contact angle of the liquid paraffin on the substrates.

Substrates	${\theta }_{E}$, º Mean ± SE	${\theta }_{R}$, º Mean ± SE
Glass	17.9 ± 1.0	5.0 ± 1.6
Silicon	35.1 ± 0.4	32.9 ± 1.7
methacrylate low-viscosity resin	31.1 ± 1.3	24.1 ± 2.5
polystyrene EOG	3.3 ± 0.4	0.86 ± 0.86
Polystyrene	7.7 ± 0.4	3.1 ± 0.6
Sapphire	11.8 ± 0.7	6.3 ± 1.6

${\theta }_{E}$ is the contact angle of the liquid at equilibrium and ${\theta }_{R}$ is the receding contact angle.

### Observation of the contact area of setal terminals and secretory fluid on substrates

Individual adult ladybird beetles were placed in a small square polystyrene case in order to observe the contact area of individual setal terminals of freely walking beetles. The adhesive pads of the beetles were observed through the plastic case by using an inverted microscope (Eclipse Ti, Nikon, Tokyo, Japan). Tarsi and tenent setae were observed by using a scanning electron microscope (LEO1550, Carl Zeiss NTS GmbH, Oberkochen, Germany).

### Observation of the secretory fluid distribution between setae and substrates

The secretory fluid between individual pad setae on the substrate was observed by cryo-SEM on an Hitachi S-4800 microscope (Hitachi High Technology, Tokyo, Japan) equipped with a Gatan Alto 2500 cryo-preparation system (Gatan, Pleasanton, CA, USA). The tarsus was cut off the leg of the living anesthetised beetle and gently placed in contact with the glass substrate (Soda-lime glass (Pre-cleaned cover slips, diameter 10 mm, order number 631–0170, Chemfidence, Frankfurt am Main, Germany)) with and without a thin film (10 nm) of AuPd. The surface roughness of a glass substrate with an AuPd thin layer was estimated by atomic force microscopy (AFM L-trace, SII NanoTechnology, Tokyo, Japan). The sample was cooled rapidly in liquid nitrogen and then placed on a cryo-stage which was previously cooled to − 140 °C in the microscope. The frozen secretory fluid was then observed in the contact area between the setae and substrate by cryo-SEM at − 120 °C with an accelerating voltage of 3 kV. After observation of the setae on the substrate, the tarsus with the setae was mechanically removed from the substrate in the cryo-preparation chamber, and the residues of the frozen fluid in contact with the substrate were further observed.

### Traction force of beetles on various substrates

A load cell/force transducer with a 245 mN capacity (World Precision Instruments, Sarasota, FL, USA) was clamped to a holder perpendicular to the horizontal substrate surface. 12 females and 12 males were used for the experiment. The beetles were anaesthetised with carbon dioxide. Each beetle was weighed after 1 h of recovery from anaesthesia. Each beetle was then attached by its dorsal surface to a human hair by using a drop of molten beeswax. The free end of the human hair was mechanically attached to the force transducer. When walking, the tethered beetle pulled the force transducer via the attached hair. Each beetle was tested once on each different surface in a random order. The force data were recorded digitally with the BIOPAC System MP100 and software ACQ Knowledge 3.7.3 (World Precision Instruments, Inc., Sarasota, FL, USA), and the maximum pulling force during a 60 s pulling period was estimated for each trial.

### Pull-off forces between the polymer structures and various substrates

Ten polymer structures comprising a thin plate on the top of specially-shaped pillars were made of polydimethylsiloxane (PDMS) to study adhesion with and without a fluid layer in the contact area, as described previously^20^. Such a microstructure mimics the discoid adhesive setae of male chrysomelid and coccinellid beetles^21^. Our structured polymer is shown in the inset of Fig. 4a. A thin liquid layer of Paraffine oil (density (20 °C) 0.815–0.840 g mL^−1^, kinematic viscosity (37.8 °C) 8.8–11.0 mm^2^ s^−1^, Wako Pure Chemical Industries) was used to cover the top of polymer structure as follows. Liquid paraffin (16.8 µL) was dropped onto a glass Petri dish with an inside diameter of 106 mm and spread over the whole glass surface by a nitrogen jet. This amount of liquid on the glass Petri dish is comparable to a liquid thickness of 1.9 µm. The structured side of the polymer was attached to the Petri dish, and the thin liquid layer was transferred onto the structured polymer surface. Because of the main component of the secretory fluid of seven spotted ladybird beetle is 9-Methylheptadecane^11^, which is a type of alkane, paraffin oil, a type of alkane, was used in the experiment.

Pull-off forces were measured using a digital force gauge with a 50 N capacity (ZP 50 N, IMADA Co., Ltd., Aichi, Japan) and were recorded using the software ZP-recorder ver.2.11 (IMADA Co., Ltd., Aichi, Japan). Five polymer structures were prepared for the experiment. The structures were attached to the substrate and were pulled at a rate of 300 mm min^−1^. The experiment was repeated five times per a structure with each substrate for both dry samples and those covered with liquid paraffin. Liquid paraffin oil was re-applied after each trial by re-attachment to the glass Petri dish containing the liquid paraffin.
